# Defective Interfering Particles of Influenza Virus and Their Characteristics, Impacts, and Use in Vaccines and Antiviral Strategies: A Systematic Review

**DOI:** 10.3390/v14122773

**Published:** 2022-12-12

**Authors:** Min Wu, Entong Zhou, Rui Sheng, Xiaoshu Fu, Jiemin Li, Chunlai Jiang, Weiheng Su

**Affiliations:** 1 National Engineering Laboratory for AIDS Vaccine, School of Life Sciences, Jilin University, Changchun 130012, China; 2 Key Laboratory for Molecular Enzymology and Engineering of the Ministry of Education, School of Life Sciences, Jilin University, Changchun 130012, China

**Keywords:** defective interfering particles, defective viral genomes, influenza virus, viral replication, interferon, NS1, live attenuated influenza vaccine, clinical trial, antiviral

## Abstract

Defective interfering particles (DIPs) are particles containing defective viral genomes (DVGs) generated during viral replication. DIPs have been found in various RNA viruses, especially in influenza viruses. Evidence indicates that DIPs interfere with the replication and encapsulation of wild-type viruses, namely standard viruses (STVs) that contain full-length viral genomes. DIPs may also activate the innate immune response by stimulating interferon synthesis. In this review, the underlying generation mechanisms and characteristics of influenza virus DIPs are summarized. We also discuss the potential impact of DIPs on the immunogenicity of live attenuated influenza vaccines (LAIVs) and development of influenza vaccines based on NS1 gene-defective DIPs. Finally, we review the antiviral strategies based on influenza virus DIPs that have been used against both influenza virus and SARS-CoV-2. This review provides systematic insights into the theory and application of influenza virus DIPs.

## 1. Introduction

Defective interfering particles (DIPs) are defective viral genome (DVG)-containing particles generated during viral replication. DIPs have been found in many viruses, especially RNA viruses [1,2,3]. In 1954, influenza virus was first reported to contain DIPs [4]. Many negative-strand RNA viruses, such as the measles virus [5], mumps virus [6], rubella virus [7], rabies virus [8], Sendai virus [9], and vesicular stomatitis virus [10], generate DIPs. Although the potential mechanisms for DIPs generation are yet to be elucidated, it can be considered as a result of the low fidelity of RNA-dependent RNA polymerases (RdRps) in RNA viruses, which causes mutations and deletions, leading to defective genomes [1,11]. The form of DVG can be divided into the following categories: (1) the most common type are DVGs with single internal deletion, or some particular types with multiple internal deletions; (2) DVGs with mosaic genomes, including both deletions and insertions, and (3) DVGs with copy-back genomes [12]. Since DIPs lack at least one intact gene-expression product, e.g., polymerase, which is vital to viral life cycle, they usually need to co-infect the host with wild-type (WT) viruses, also called as standard viruses (STVs), as “helpers” to express all viral proteins and package progeny DIPs [2,11,13].

Multiple studies have shown that DIPs can interfere with STV replication in co-infected host cells. Two potential mechanisms have been suggested for this process [11,14]. First, because DVGs are shorter than the full-length STV genomes, they can replicate faster and compete for polymerases. Second, DVGs competitively scramble STV structural proteins and other viral proteins necessary for the encapsulation of virion particles, resulting in a notable decrease in STV progeny. Additionally, DIPs can initiate stronger innate immune responses by stimulating the synthesis of interferons (IFN). DVGs can be easily recognized by pattern recognition receptors (PRRs), including retinoic acid-inducible gene I (RIG-I), laboratory of genetics and physiology 2, and melanoma differentiation–associated gene 5 [6,15,16,17]. The stimulation of PRR signaling induces the expression of type I IFNs and several proinflammatory cytokines, all of which play key roles in dendritic cell maturation and the regulation of adaptive immunity [18]. Because of these characteristics, DIPs have become a concern in various live attenuated vaccines including influenza virus [19], measles virus [5], rubella virus [7], mumps virus [6], and rabies virus [8]. Additionally, DIP-based antiviral strategies have been widely explored to treat various infections due to viruses, such as SARS-CoV-2 [20], influenza virus [21,22], and enteroviruses [23].

Influenza viruses are broadly prevalent respiratory pathogens that cause severe public health problems globally. Influenza virus DIPs and DVGs are of special concern because the influenza virus genome is composed of segmented RNAs. This review summarizes the characteristics and occurrence of influenza virus DIPs and their roles in interfering with viral replication and stimulating the innate immune response. Furthermore, the impact of DIPs in LAIVs and DIP-based LAIVs, as well as the antiviral strategies based on influenza virus DIPs, are discussed.

## 2. Virological Characteristics of Influenza Virus DIPs and DVGs

Influenza viruses have always been a major threat to human health. The prevalence of seasonal influenza increases every few years, and global influenza pandemics occur irregularly [24]. The World Health Organization estimates that 290,000–650,000 people die each year from respiratory diseases related to seasonal influenza, and effective control of influenza virus remains elusive [25].

Influenza virus can be divided into four types: A, B, C, and D. Influenza A virus (IAV) often causes serious disease and is further divided into subtypes based on the antigens hemagglutinin (HA) and neuraminidase (NA), such as H1N1 and H3N2 [26]. The influenza virus can also be classified according to the infected host, including human influenza, avian influenza, and swine influenza virus. Because of the high proportion of DIPs in influenza virus, systematic studies of DIPs are indispensable for understanding its behavior.

### 2.1. Mechanisms Underlying Generation of Influenza Virus DIPs

**Figure 1:**
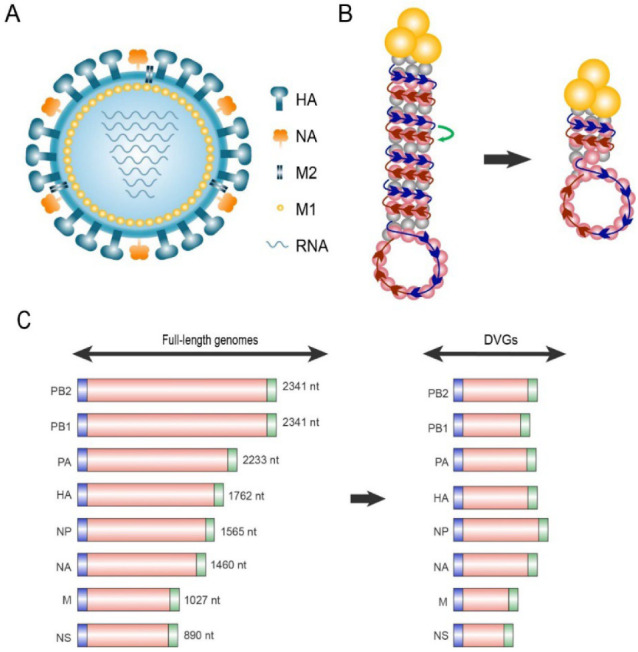
Schematic diagram of standard viruses (STV) and defective interfering particles (DIPs). (**A**) Schematic structure of the influenza A virus. (**B**) Mechanisms underlying the generation of influenza defective viral genomes (DVGs). The ribonucleoprotein consists of RNA-dependent RNA polymerase (RdRp, yellow trimer) and the single-stranded vRNA (−) genome coated with nucleoproteins. The RdRp, which originally synthesizes cRNA (+) along the vRNA (−) template (blue arrows) during replication, erroneously translocates to the adjacent antiparallel chain vRNA (−) (red arrow) in the RNP, causing large-scale deletion of the intermediate skipped sequences in DVGs. (**C**) Schematic length of full-length genomes and DVGs. Both full-length RNAs and DVGs contain 3′ (blue) and 5′ (green) terminal packaging signal sequences.

The influenza virus belongs to the Orthomyxoviridae family and is composed of a lipid membrane, a layer of matrix proteins, and a segmented, single-stranded, negative-sense RNA genome (Figure 1A). The viral genome comprises eight segments of nucleoprotein (NP)-wrapped RNA [27]. Segments 1–3 encode RNA polymerases, namely PB2, PB1, and PA, responsible for RNA transcription and replication. Segments 4 and 6 encode two surface glycoproteins, HA, and NA, respectively. Segment 5 encodes NP, which is responsible for assembly of the viral RNA. Segment 7 encodes matrix protein M1, which consists of the matrix protein layer beneath the viral envelope, and ion channel protein M2 embedded in the viral envelope. Segment 8 encodes the non-structural proteins 1 (NS1), whose functions are mainly antagonizing host antiviral reactions, and NEP/NS2, the nuclear export protein.

Each negative-strand viral RNA (−) segment is assembled in a complex named viral ribonucleoprotein (vRNP) consisting of a heterotrimeric RdRp and NPs (Figure 1B). In the vRNP, most vRNA (−) regions are coated with NPs, such as the histones, forming an anti-parallel double helix. One end of the double helix contains the 5′ and 3′ termini of vRNA (−), which bind to the RdRp heterotrimer comprising PB2, PB1 and PA. Meanwhile, the other end forms a loop where the NP-coated vRNA (−) chain turns back [28]. One possible generation mechanism of influenza virus DVGs may be the erroneous translocation of RdRp in the helical structure of ribonucleoproteins (RNP). RdRp, which originally transcribes in the correct direction of the vRNA (−) during replication, translocates to the adjacent vRNA (−) in the antiparallel direction for unknown reasons, and transcribes the remaining sequence. Thus, the intermediate skipped sequences are missing from the cRNA (+) product, producing DVGs [11,28,29,30].

### 2.2. Genomic Characters of Influenza Virus DVGs

In addition to DVGs, viral RdRp generates different aberrant RNA species during influenza virus replication, such as mini viral RNAs (mvRNAs) and small viral RNAs (svRNAs) [31,32,33,34]. Jennings et al. speculated that DVGs are longer than 178 nt and can form the RNP structure [29]. Turrell et al. reported that mvRNAs are shorter than 149 nt and contain both the 5′ and 3′ termini of vRNAs [35]. Perez et al. showed that svRNA are 22–27 nt long and contain only the 5′ end of vRNA segments [36]. Both mvRNAs and svRNAs fail to form RNPs [31,34].

According to a large number of studies on influenza virus DIPs, the particles have distinct characteristics, including style, length, and deletion site of DVGs. The most prevalent influenza virus DVG-style is a single internal deletion within a segment [29]. The length of multiple DVGs of influenza virus ranges from 200 to 800 nt (Figure 1C) [37]. In segments 1, 2, and 3, the average final DVG length is 400–500 nt, indicating that approximately 1800–1900 nt are missing compared to full-length genomes [38,39]. In segments 4–8, the average length of DVGs is 400 nt [40,41]. Notably, all influenza virus DVG segments contain a generic bipartite packaging signal including 12–13 nt at the 5′- and 3′-terminals that can be identified as vRNA (−) for assembly into RNPs [42]. Sequencing of both clinical and cell-cultured influenza virus samples revealed that DVGs retain at least 50–150 nt sequences at both the 5′- and 3′-termini [38,43], suggesting that influenza DVGs can miss most sequences outside the packaging signal.

The DVGs of influenza virus are mainly generated from polymerase segments, including segments 1 to 3 [38,39]. The DVGs from non-polymerase segments are relatively low in abundance and their capacity to interfere with STV replication, or to induce an interferon response, is rarely reported, except segment 8 encoding NS (discussed in Section 2.4 and Section 3.2). DVGs produced by different influenza virus strains vary in their characteristics (Table 1). For example, a H1N1 strain isolated from a 23-year-old man contained DVGs in segments 1 and 3, whereas another H1N1 strain isolated from a 35-year-old woman at the same location contained DVGs in segment 3 only [37,44]. Multiple DVGs were detected in segments 1, 2, and 3 in six Chinese patients (age: 10–76 years old) with H7N9 infection in Hong Kong during 2014–2017, whereas the percentage of DVGs was very low, or possibly zero, in four H3N2-infected Chinese patients in Hong Kong during 2017 [39].

Moreover, the DVGs produced by the same influenza virus strain may differ in various types of infected cells in vitro. In strain A/Puerto Rico/8/34 grown in embryonated eggs, most DVGs were derived from segments 1, followed by segments 2–6 and 8 [29]. However, only A/PR/8/34 DVGs of segments 1–3 and 6 were detected in infected MDCK cells, regardless of whether the multiplicity of infection (MOI) was high (1) or low (0.00025) [40].

In addition, the DVGs produced by the same influenza virus strain may differ between in vitro and in vivo infection. DVGs of A/WSN/1933 (H1N1) were generated from segments 1–6 in the allantoic fluid of embryonated eggs, whereas DVGs from segments 1–3 and 7 were only detected in the lungs of mice [45]. Similarly, DVGs from segments 1–8 of A/equine/Newmarket/7339/79 (H3N8) in the allantoic fluid of embryonated eggs were observed, whereas those from segments 1–4 or 1–6 only were noted in the lungs of mice [41]. These data suggested that the host tissue (mouse lung) was selective for a subset of inoculum DVGs. Table 1 summarizes the data on existing segment DVGs in different IAV strains.

### 2.3. Interfering Effects and Underlying Mechanisms of Influenza Virus DIPs

There are several proposed mechanisms for how influenza virus DVGs interfere with STVs replication. First, since the DVG is shorter than the STV in length and is quicker to replicate, the proportion of STV is gradually reduced during co-infection. Second, since influenza viral RNA exists and replicates in the form of RNPs, DVG competes with RdRps and NPs to encapsulate its own vRNA (−) and replicate, reducing the proportion of RNPs obtained by STV vRNA (−). Third, DVG RNPs can also compete with the RNPs, structural proteins, and glycoproteins of other STV segments to package DIPs [14,46]. Theoretically, the presence of DIPs can reduce the effective amount of STVs, resulting in lower infectious virus yield. In a study by Frensing et al., two seeds of the influenza strain A/PR/8/34 containing high and low proportions of DIPs were produced. After infecting MDCK cells at the same MOI, the titer of progeny STV (determined by TCID_50_ [tissue culture infectious dose to infect 50% of cells in a culture]) produced at 16 h post-infection (hpi) in the high proportion of DIP group was 1000-fold lower than that in the low proportion of DIP group [40]. Moreover, the ability of DIP to interfere with STV replication is closely related to the MOI. Isken et al. compared the total virion (determined by HA) and infectious titer obtained for different MOI. They found that there was no significant difference in total virions among different MOI. However, the proportion of infectious virion decreased sharply with an increase in MOI, whereas the proportion of DIPs increased [47]. A reasonable explanation is that when MOI increased, the chance of STV and DIP co-infecting the same cell was also increased. Since DIPs are more competitive than STVs during replication, a large number of DIPs are produced, and the number of STVs replicates is reduced.

In addition, some studies have shown that infection by high levels of DIPs can accelerate apoptosis, which contributes to the lower virus yield [40,48]. The presence of a high proportion of DIPs also reduces the pathogenicity of the influenza virus. Rabinowitz & Huprikar revealed that the symptoms of IAV-challenged mice with a high proportion of DIPs were less severe than those with a low proportion of DIPs [48]. Scott et al. identified an IAV DIP containing a large deletion in the *PB2* gene segment that provided broad-spectrum antiviral activity. This DIP exerted protection in mice against several different subtypes of IAV, suggesting that replication competition provides homologous protection [49]. Furthermore, Vasilijevic et al. analyzed the samples of clinical patients with IAV infection and found that patients with less accumulation of DVGs had a higher risk of death than those with more, suggesting that high proportions of DIPs reduce viral infectivity and pathogenicity in vivo [37]. In addition, DVGs containing a specific *NS1* gene deletion can cause a reduction in STVs titer through both competitive replication and inactivation of IFN antagonism. Chen et al. reported that the R38A/K41A mutation in the *NS1* gene caused the loss of IFN-β antagonism of the innate immune response, resulting in failure of IAV replication in normal cells after three to six generations [50]. Compared to that in the wild-type (WT) influenza A/PR/8/34, virus titer was significantly reduced in the DVG group containing an *NS1* gene deletion, with viral titer 1000-fold lower in MDCK cells and 10-fold lower in Vero cells at 72 hpi [51]. Additional functions of DVGs with *NS1* gene deletion are discussed in Section 2.4.

### 2.4. Immune Activation and Underlying Mechanisms of Influenza Virus DIPs

DVGs can promote stronger innate immune responses than STVs, in particular via the promotion of type I IFN expression, resulting in a stronger antiviral response and further inhibiting STV replication. Scott et al. reported that in immunodeficient mice, DIPs could delay influenza symptoms for a short period when co-infected with IAV [48]. In contrast, DIPs can continuously suppress IAV without symptoms in wild mice, suggesting the important role of DIPs in immune activation [48].

DVGs stimulate robust innate immune responses, especially the synthesis of IFN type I. Bdeir et al. reported that the expression levels of *MxA*, an IFN-stimulating gene, was increased by approximately 10,000-fold and 1000-fold in Calu-3 cells when infected with DIPs, which harbored deletions in the PB2 or PA gene segment, respectively [52]. In a low-proportion (76.58%) DIP group, the IFN-β expression level was 37-fold higher than that in a high-proportion (99.78%) DIP group in infected MDCK cells at 8 hpi [40]. Ayaz et al. showed that the high-proportion (66.5%) PA DVG group induced a three-fold increase in IFN-β mRNA levels compared to the low-proportion (17.5%) PA DVG group, resulting in STV titer loss in primary human nasal epithelial cells [53]. When mice were challenged with IAV stocks containing a high proportion of DVGs or lacking DVGs, there was no significant difference in the total amount of replicated IAV genome in the first 3 days post-infection (dpi) between the two groups [54]. However, the relative copies of IFN-β mRNA were increased approximately two-fold in the high-proportion DVG group, and their symptoms were relieved [54]; these phenomena are basically consistent with the effect of Sendai virus DVGs [54]. However, not all cell types can trigger an immune response in the presence of DVGs; the extent of activated immune response in infected cells is different owing to the heterogeneity of DVG accumulation [55,56]. Activation of the innate immune response can further trigger systemic immunity. In other words, DVGs act as an adjuvant [18,57].

The key to innate immunity activation by DVG RNA molecules is their strong activation of PRRs. RIG-I plays a critical role in the recognition of influenza virus genomic RNA [58]. RIG-I-specific pathogen associated molecular patterns are characterized mainly by RNA molecules with a 5′-triphosphate (5′-ppp) group and partial blunt-ended double-stranded RNA composition [59,60,61,62]. Baum et al. explored the characteristics of influenza virus and Sendai virus RNA that are recognized by RIG-I using next-generation sequencing [17]. Sendai virus is a negative-sense, single-stranded RNA virus belonging to the Paramyxoviridae family; both the influenza virus and Sendai virus contain a 5′-ppp group in their genome RNA. Only the copy-back form DVG of the Sendai virus (546 nt) specifically interacted with RIG-I, not the full-length viral genome RNA (15,384 nt) [17]. RIG-I interacts with all influenza virus RNA segments but preferentially interacts with shorter RNA molecules. These shorter viral RNA molecules include NP (1565 nt), M (1027 nt), truncated NS (approximately 418 nt) gene, and DVGs (ranging from approximately 200–800 nt) generated from larger PB2, PB1 and PA gene segments [17]. These results imply that DVGs always trigger stronger innate immune responses than the full-length viral genome.

In addition to DVGs, mvRNAs are ligands for RIG-I and have reserved panhandle structures with closely apposed 5′ and 3′ ends. They can probably initiate antiviral signal transduction [31]. Kato et al. found that mvRNAs induced significantly higher IFN expression than the full-length viral genome [63]. Activation of RIG-I causes it to associate with the mitochondrial antiviral signaling protein (MAVS) on the outer surface of the mitochondria, followed by activation of the transcription factors interferon regulatory factor 3 and nuclear factor-kappa B, which promote the transcription of type I IFN and other proinflammatory cytokines, causing a subsequent antiviral response and adaptive immunity. Besides DVGs, Boergeling et al. reported that a Thailand/1(KAN-1)/2004 (H5N1) segment 2 DVG-translated protein, PB2_Δ_, induces the expression of IFN-β and IFN-stimulated genes by direct interaction with MAVS; such effect is independent of the existence of defective viral RNA [64]. The PB2_Δ_ protein can consequently reduce viral replication of IAV or vesicular stomatitis virus.

The unique function of the influenza virus protein NS1 as an IFN antagonist is discussed separately. NS1 was encoded by the IAV genome segment 8, and binding of dsRNA with IAV NS1 prevents RIG-I-mediated detection of defective viral RNAs [33,65]. NS1 can also bind to tripartite motif-containing protein 25, also known as estrogen responsive finger protein, which acts as a ubiquitin E3 ligase and inhibits the downstream pathway of RIG-I [65]. NS1 also inhibits IFN expression at the post-transcriptional level by binding to the smallest components of cleavage and polyadenylation specificity factor that catalyzes the addition of a poly-A tail to IFN mRNA [65]. However, these inhibitory effects are strain-specific [66]. DVGs generated from segment 8, especially those missing a part or the entire NS1 gene-coding sequence, lose their antagonistic activity against IFN. IFN expression was elevated in A549 and HEK293 cells infected with DIPs containing *NS1* gene deletion DVGs compared with those infected with WT IAV [51,67]. Compared with WT influenza B virus (IBV), the NS1 gene-truncated IBV induced remarkably higher levels of cytokines, including IFN type I, TNF-α, IL-6, and IL-1β, in both macrophages and human nasal epithelial cells [68]. Ferko et al. reported that a DIP containing truncated *NS1* gene DVGs with a deletion at the 5′ end and a length of 40–80 nt not only elicited markedly higher levels of type I IFNs in the serum of mice than the WT IAV, but also stimulated high expression of cytokines, such as IL-1β and IL-6 [69]. Based on these results, DVGs with an *NS1* gene deletion, or DIPs containing DVG with an *NS1* gene deletion, are believed to have the characteristics of attenuated vaccines with antiviral functions (further discussed in Section 3.2 and Section 4).

### 2.5. Significance of DIPs in Persistent Infection of Virus Population

DIPs may act as “modulators” during viral infection to avoid premature apoptosis of host cells caused by excessive accumulation of STV, thus promoting persistent viral infection [2,34]. DIPs are suggested to contribute to the establishment of persistent infections in various RNA viruses, such as influenza virus [70], respiratory syncytial virus [71], measles virus [72], and Ebola virus [73]. Sidhu et al. found that measles DVGs can accumulate in the brain cells of patient with subacute sclerosing panencephalitis for a long time in some rare cases [74]. DVGs were also detected during the persistence of Ebola virus infection in tissue cultures [75]. Pelz et al. revealed periodic oscillations of DIPs and STV titers during infection in vitro [43]. Moreover, Rüdiger constructed a multiscale model of DIP replication during IAV infection in MDCK cells and suggested that a high proportion of DIPs can prevent apoptosis induced by IAV acute infection [76]. However, knowledge on the contribution of DIPs to persistence in vivo is limited. In addition, how DIPs affect the quasi-species structure of viral population and alter their fitness and transmissibility remains be explored.

## 3. Potential Impact of DIPs on LAIV Immunogenicity, and the Development of DIP-Based LAIVs

### 3.1. Potential Impact of DIPs on Immunogenicity of LAIVs

LAIVs are an important part of influenza prevention owing to their efficiency and lasting immunogenicity in inducing humoral, cellular, and mucosal immunity, and the advantages of a nasal spray. Given that DIPs are extensively found in influenza virus, they also exist in commercial LAIVs. Because DIPs suppress STV viral replication and stimulate a strong innate immune response, they likely have important and complex effects on immunogenicity and the protective effect of conventional LAIVs (these differ from recombinant DIP-based LAIVs, discussed in Section 3.2). However, these effects are still largely unknown. Currently, the proportion of DIPs in LAIV products is not a quality control index for any manufacturer in any country. The discovery of impact of DIPs on the immunogenicity and protectivity of LAIVs will also promote the improvement of commercial LAIVs.

DIPs are predominantly found in commercial LAIVs. Our assays on seed viruses for commercial LAIVs showed that the DIP proportions of total particles were 92.7% for A/New-York/61/2015-CDC-LV16A (H1N1), 99.5% for A/17/HongKong/2014/8296 (H3N2), and 98.0% for B/56/Brisbane/60/08 (Victoria) based on total viral particle (hemagglutination assay) and infectious virus (50% egg infection dose) titration (unpublished data). Gould et al. examined the European version of LAIV FluMist^®^ (Fluenz™ Tetra) produced in the 2014–2015 vaccine season, which consists of A/California/7/2009 (H1N1), A/Texas/50/2012 (H3N2), B/Massachusetts/2/2012 (B/Yamagata lineage), and B/Brisbane/60/2008 (B/Victoria lineage) [77]. The group detected a large number of DVGs in segments 1–3 (with DIPs estimated to make up 99.75% of the total particles) [19]. FluMist^®^ was withdrawn by the Centers for Disease Control and Prevention (CDC) during the 2016–2017 flu season in the USA because of its low effectiveness between 2013 and 2016. The large amount of DVGs in LAIVs may be an important reason for the reduced effectiveness of FluMist^®^, apart from prevalent strain prediction bias [19]. Another study by Ayaz et al. examined DVGs in 11 historical H1N1 LAIV strains using digital RT-PCR [53]. DVGs in segments 1–3 were detected in all 11 H1N1 LAIV strains tested; DVGs from PA made up the highest proportion (between 10.2% and 27.8% in different strains) of DVGs from any segment. This study also indicated that a higher proportion of PA DVGs does not impair the replication of the full-length viral genome in two strains [53].

Generally, nucleic acid-based analytical measurements, including PCR and next-generation sequencing, can underestimate the level of DVGs; thus, particle-based or titer-based measurements are widely used owing to their superior precision [40,78]. Scientifically, these sorts of studies should be reproduced. Stimulation of the innate immune response by DIPs can promote the adaptive immune response, which could benefit the immunogenicity of LAIVs. Thus, LAIV immunogenicity is a balance between the depression of full-length virus levels and elevation of the innate immune response. DIPs may contribute to the attenuation of LAIV virulence. In summary, the proportion of DIPs in the total particles of LAIVs is crucial for maintaining balanced immunogenicity. Further studies on how DIPs affect immunogenicity and the protective effects of conventional LAIVs are urgently needed.

### 3.2. Development of LAIVs Based on NS1 Defective Genome

In recent years, a special kind of attenuated influenza vaccine with a genome consisting of a genetically modified segment 8 with NS1 gene deletion or truncation and the other seven full-length segments has drawn wide attention (Table 2). These viruses are replicable, although defective in genome, because NS1 is not vital for viral replication; however, their progeny virus yields are largely restricted due to the loss of IFN antagonist function, which corresponds with the character of attenuated vaccine [79,80,81,82]. Further, NS1 gene deletion or defect induces higher IFN expression and promotes strong innate and subsequent adaptive immune responses, leading to high immunogenicity [69,83,84].

Ngunjiri et al. [85] and Ghorbani et al. [86,87] developed the NS1 gene truncated A/TK/OR/71 (H7N3) LAIVs (mutant pc2 and pc4). pc2-LAIV protected mice against a heterologous virus strain A/CK/NJ/150383–7/02 (H7N2) challenge by preventing body weight loss and reducing viral shedding [85]. pc2-LAIV and pc4-LAIV induced the generation of neutralizing antibodies in pigs and protected pigs against challenge with swine-origin A/TK/OH/313053/04 (H3N2) by reducing viral shedding [85]. pc4-LAIV also induced the generation of virus-specific antibodies and neutralizing antibodies in chickens, and reduced virus shedding in the trachea when challenged with A/chicken/PA/13609/93 (H5N2) in chickens [86]. Further, Ghorbani et al. improved pc4-LAIV by combination with mutations NS1 Δ76-86 and PB2-D309N, which enhanced the expression of IFN and IFN-related genes in chick embryo fibroblast cells and chickens [87]. This vaccine also reduced virus shedding in chickens after challenge with heterologous A/chicken/NJ/150383-7/02 (H7N2) [87]. Rathnasinghe et.al demonstrated that pre-treatment with a LAIV lacking the NS1 gene coding sequence (ΔNS1) 8 or 24 h before lethal infection by a highly virulent A/PR/8/34 (H1N1) influenza virus can prevent weight loss in mice in a dose-dependent manner [88]. Wang et al. revealed that the CA4-DelNS1 (H3N2) LAIV containing an NS1 gene deletion was highly attenuated in mice and induced not only cross-reactive broad-spectrum neutralizing antibodies, but also CD8+ and CD4+ T cell immunity [79]. Lee et al. reported that intradermal vaccination of the CA4-DelNS1 (H1N1) LAIV containing an NS1 gene deletion protected mice against not only homologous H1N1 influenza virus, but also heterologous H7N9 and H5N1 influenza challenges. These protections lasted for 6 months after immunization [80].

NS1 gene deletion-based LAIVs have also shown promising immunogenicity in clinical trials (Table 2). Wacheck et al. showed that a ΔNS1-H1N1 vaccine lacking the complete NS1 open reading frame was produced in Vero cells [89]. A total of 48 volunteers were intranasally immunized with different doses of ΔNS1-H1N1 vaccine. It was well tolerated and did not appear to cause severe adverse events, suggesting the safety of this vaccine. Shedding of vaccine virus could not be detected in subject nasal washings more than 12 h after immunization. Furthermore, the highest dose group had significantly increased vaccine-specific mucosal IgA, serum IgG, and neutralizing antibodies. Importantly, the vaccine induced cross-neutralizing antibodies against heterologous IAV strains [89].

The same group conducted a subsequent phase I/II trial of a trivalent delNS1 vaccine containing H1N1, H3N2, and B strains [82]. The virus was not detected in volunteers’ nasal lavage fluid at 24 hpi, and adverse symptoms were not observed in the vaccine group, proving the safety of the vaccine. This trivalent delNS1 vaccine can also induce significant levels of broad-spectrum neutralizing antibodies [82]. Nicolodi et al. conducted a phase I study in 36 healthy adults who received two intranasal immunizations of live attenuated H5N1 vaccine (delNS1-H5N1) lacking an NS1 coding sequence [90]. Only mild adverse symptoms were noted within 7 days after the first and second immunization, and no vaccine viral shedding was observed at any time point, suggesting vaccine safety and tolerance [90]. A serum neutralizing antibody response against homologous H5N1 strain was detected in 75% of volunteers in the high-dose group after the first immunization; this increased to more than 90% after the second immunization. Vaccine-specific nasal IgA antibodies were observed in 17.0% of volunteers in the high-dose group after the first immunization; this increased to 41.7% after the second immunization [90]. The novel strategy of basing LAIVs on NS1 gene deletion or defection DIPs can provide promising influenza vaccine candidates.

**Table 2:** Preclinical and clinical LAIVs based on NS1 gene deletion or defection DIPs.

Name	Defection in Genome	Strain, Subtype	Preclinical or Clinical	Immunogenicity and Protection	Safety	Reference
NS1-truncated mutants pc2	Deletion of 370 to 426 nt in NS1 coding region	A/TK/OR/71 (H7N3)	Preclinical, in mice and pigs	Highly neutralizing antibodies; strong protection without weight loss in both mice and pigs	Highly attenuated	[85]
NS1-truncated mutants pc4	Deletion of 301 to 492 nt in NS1 coding region	A/TK/OR/71 (H7N3)	Preclinical, in pigs and chickens	Highly neutralizing antibodies in pigs and chickens; strong protection without weight loss in pigs	Highly attenuated	[85,86]
ΔNS1	Deletion of the NS1 coding region	Reassortant 25A-1 (H1N1) containing the NS gene segment from the cold adapted strain A/Leningrad/134/47/57 (H1N1), and remaining gene segments from A/Puerto Rico/8/34 (H1N1)	Preclinical, in mice	Strong protection without weight loss	Highly attenuated	[88]
CA4-DelNS1 (H3N2)	Deletion of the NS1 coding region	HA and NA gene segments derived from A/Hong Kong/4801/2014 (H3N2) and remaining gene segments from A/California/04/2009 (H1N1); cold adapted	Preclinical, in mice	Highly cross-reactive broad-spectrum neutralizing antibodies and CD8+ and CD4+ T cell immunity; strong protection without weight loss	Highly attenuated	[79]
CA4-DelNS1 (H1N1)	Deletion of the NS1 coding region	A/California/04/2009 (H1N1); cold adapted	Preclinical, in mice	Highly cross-reactive broad-spectrum neutralizing antibodies and CD8+ and CD4+ T cell immunity; strong protection without weight loss	Highly attenuated	[80]
ΔNS1-H1N1	Deletion of the NS1 coding region	HA and NA gene segments derived from A/NC/20/99 (H1N1), and remaining gene segments from the reassortant IVR-116 (H1N1)	Clinical phase I	Induction of specific mucosal IgA and cross-neutralizing antibodies	No severe adverse events and no viral shedding after 12 hpi	[89]
Trivalent delNS1 vaccine	Deletion of the NS1 coding region	HA and NA gene segments derived from A/Brisbane/59/07 (H1N1), A/Brisbane/10/07 (H3N2), or B/Florida/04/06, and NS gene segment from A/Puerto Rico/8/34 (H1N1)	Clinical phase I/II	Induction of broad-spectrum neutralizing antibodies	No severe adverse events and no viral shedding at 24 hpi	[82]
delNS1-H5N1 vaccine	Deletion of the NS1 coding region	A/Vietnam/1203/04 (H5N1)	Clinical phase I	Induction of specific mucosal IgA and significant neutralizing antibodies	No severe adverse events and no viral shedding	[90]

## 4. Antiviral Strategies Based on Influenza Virus DIPs

### 4.1. Antiviral Activity of Influenza Virus DIPs against Influenza Virus

Owing to the properties described above, DIPs can also be used as an antiviral agent to antagonize influenza infection [91]. DI244 particles, derived from influenza A/PR/8/34 and containing a deletion in the PB2 gene segment from 151 to 2098 nt, showed strong antiviral activity [79,92,93,94]. A single dose of DI244 particles completely protected mice from weight loss against challenge with influenza A/WSN/40 (H1N1) and other subtypes (H2N2, H3N2, and H3N8) [49,94]. Dimmock et al. confirmed that treatment with DI244 can protect ferrets from initial infection and re-infection with influenza A/California/04/09 (H1N1) [95]; such protection was more effective than that by oseltamivir, a clinical compound drug [96]. A system for the production of pure DI244 using a reverse genetics approach in an MDCK cell line stably expressing PB2 was established, such that STV could be excluded from the product [93]. When pure DI244 particles alone were injected, all mice survived without body weight loss, indicating non-toxicity of DI244 [21]. Pure DI244 also protected mice from lethal doses of influenza A/PR/8/34 (H1N1); however, myxovirus resistance 1 (Mx1, an interferon stimulated gene)-deficient mice were not protected, suggesting that the IFN response may be an important component of the antiviral effects of DIPs [21].

OP7, another type of DIP derived from influenza A/PR/8/34 (H1N1), was established. OP7 particles do not contain genomic deletions but carry 37 point mutations at the packaging signal, promoter, and encoded protein regions of the M gene segment, enhancing promoter capacity and impairing vRNA packaging and M1/M2 function [97,98]. OP7 particles were non-toxic to mice, while completely protecting them from a lethal dose of influenza A/PR/8/34 (H1N1) [98]. Pelz et al. found a novel DIP named top gain (de novo) derived from semi-continuous propagation of influenza A/PR/8/34 (H1N1) in MDCK cells and containing a deletion from 269 to 2201 nt in the PB2 segment. Top gain (de novo) showed stronger antiviral activity than DI244 particles in MDCK cells, reducing STV release 10-fold compared with DI244 particles [43].

Zhao et al. developed a DVG-based antiviral system, DIG-3, comprising three plasmids encoding internal deletion DVGs of segment 1, 2, or 3 of A/WSN/1933 (H1N1) and delivered by a novel delivery peptide TAT-P1 [99]. TAT-P1/DIG-3 showed significant antiviral activity against A/HongKong/415742Md/2009 (H1N1) and A/Netherlands/219/2003 (H7N7) infection in mice, exhibiting superiority compared with treatment of zanamivir [99]. Furthermore, the same research group developed DIG-4, wherein the segment 3 DVG in DIG-3 was replaced with a shorter one and packaged by an improved delivery peptide TAT2-P1; it showed stronger antiviral activity in mice [100].

### 4.2. Antiviral Activity of Influenza Virus DIPs against Other IFN-Sensitive Respiratory Viruses

Since DIPs can induce IFN expression, they are promising candidates for antiviral therapy against IFN-sensitive respiratory viruses [13]. Easton et al. showed that a single dose of DI244 (1.2 μg) completely protected mice from lethal challenge with a paramyxovirus, pneumonia virus of mice (PVM) [101]. Rand et al. analyzed the antiviral activity of DI244 and OP7 particles against severe acute respiratory syndrome coronavirus 2 (SARS-CoV-2) and found that either type of DIP could inhibit SARS-CoV-2 replication in Calu-3 cells in a dose-dependent manner [22]. IFN-β and IFN-λ3 secretion was increased after treatment with DI244 or OP7, suggesting that the IFN response may be responsible for inhibition against SARS-CoV-2 infection [22].

However, there are safety concerns associated with DIP-based antiviral strategies, including the potential of novel hybridized virus formation if artificial DVGs recombine with a co-infectious wildtype viral genome. As DIPs are usually pre-administered, or administered simultaneously with virus challenge, there is a potential difficulty in choosing treatment timepoints in clinical use. Thus, there are still many obstacles between researchers and the clinical application of DIPs for antiviral purposes.

## 5. Conclusions and Future Directions

DIPs consist of a major proportion of influenza virus particles, and harbor two properties: interfering with the replication of standard viral genomes and eliciting an innate immune response. These properties have crucial impacts on both influenza virus infection and the immunogenicity of LAIVs. Currently, influenza virus DIPs are being developed as antiviral strategies against not only influenza, but also multiple viruses. However, many questions remain to be addressed. At present, there is no experimental method that can accurately identify and quantify DIPs and DVGs. Owing to the potential benefit of DIPs in persistence of viral replication, do they cause virulent viruses to mild population, resulting in a more widespread transmission? Furthermore, due to the two opposing effects of DIPs, limiting STV replication and triggering innate immunity, what is the integrated effect of the high proportion of DIPs on LAIV immunogenicity? Ultimately, as a commercial vaccine, does the proportion of DIPs in LAIV need to be controlled? Theoretical and practical studies are needed to uncover the importance of DIPs and utilize them in vaccine designs and antiviral agents.

## Figures and Tables

**Table 1:** Previously reported defective viral genome segments in different influenza virus A strains from different hosts.

Strain	Origin	DVG Segment	Reference
A/San Diego/INS007/2009 (H1N1)	Human, nasopharyngeal specimens	Segment 1 mainly, segments 2 and 3	[38]
A/CastillaLaMancha/RR5661/2009 (H1N1)	Human, bronchoalveolar lavages	Segments 1 and 3	[44]
A/CastillaLaMancha/RR5911/2009 (H1N1)	Human, bronchoalveolar lavages	Segment 3 only	[44]
A/Puerto Rico/8/34 (H1N1)	Embryonated egg, allantoic fluid	Segment 1 mainly, segments 2–6, 8	[29]
A/Puerto Rico/8/34 (H1N1)	MDCK cells, supernatant	Segments 1–3, 6	[40]
A/WSN/1933 (H1N1)	Embryonated egg, allantoic fluid	Segments 1–6	[45]
A/WSN/1933 (H1N1)	Mouse, lung	Segments 1–3, 7	[45]
A/equine/Newmarket/7339/79 (H3N8)	Embryonated egg, allantoic fluid	Segments 1–8	[41]
A/equine/Newmarket/7339/79 (H3N8)	Mouse, lung	Segments 1–4, or segments 1–6	[41]

## Data Availability

Not applicable.

## References

[1] Ghorbani A., Ngunjiri J.M., Lee C.W. (2020). Influenza A Virus Subpopulations and Their Implication in Pathogenesis and Vaccine Development. Annu. Rev. Anim. Biosci..

[2] Ziegler C.M., Botten J.W. (2020). Defective Interfering Particles of Negative-Strand RNA Viruses. Trends Microbiol..

[3] Alnaji F.G., Brooke C.B. (2020). Influenza virus DI particles: Defective interfering or delightfully interesting?. PLoS Pathog..

[4] Von Magnus P. (1954). Incomplete forms of influenza virus. Adv. Virus Res..

[5] Bellocq C., Mottet G., Roux L. (1990). Wide occurrence of measles virus subgenomic RNAs in attenuated live-virus vaccines. Biologicals.

[6] Santak M., Markusic M., Balija M.L., Kopac S.K., Jug R., Orvell C., Tomac J., Forcic D. (2015). Accumulation of defective interfering viral particles in only a few passages in Vero cells attenuates mumps virus neurovirulence. Microbes Infect..

[7] Derdeyn C.A., Frey T.K. (1995). Characterization of defective-interfering RNAs of rubella virus generated during serial undiluted passage. Virology.

[8] Clark H.F., Parks N.F., Wunner W.H. (1981). Defective interfering particles of fixed rabies viruses: Lack of correlation with attenuation or auto-interference in mice. J. Gen. Virol..

[9] Sokol F., Neurath A.R., Vilcek J. (1964). Formation of Incomplete Sendai Virus in Embryonated Eggs. Acta Virol..

[10] Huang A.S., Greenawalt J.W., Wagner R.R. (1966). Defective T particles of vesicular stomatitis virus. I. Preparation, morphology, and some biologic properties. Virology.

[11] Frensing T. (2015). Defective interfering viruses and their impact on vaccines and viral vectors. Biotechnol. J..

[12] Beauclair G., Mura M., Combredet C., Tangy F., Jouvenet N., Komarova A.V. (2018). DI-tector: Defective interfering viral genomes’ detector for next-generation sequencing data. RNA.

[13] Dimmock N.J., Easton A.J. (2015). Cloned Defective Interfering Influenza RNA and a Possible Pan-Specific Treatment of Respiratory Virus Diseases. Viruses.

[14] Genoyer E., Lopez C.B. (2019). The Impact of Defective Viruses on Infection and Immunity. Annu. Rev. Virol..

[15] Shingai M., Ebihara T., Begum N.A., Kato A., Honma T., Matsumoto K., Saito H., Ogura H., Matsumoto M., Seya T. (2007). Differential type I IFN-inducing abilities of wild-type versus vaccine strains of measles virus. J. Immunol..

[16] Mura M., Combredet C., Najburg V., Sanchez David R.Y., Tangy F., Komarova A.V. (2017). Nonencapsidated 5′ Copy-Back Defective Interfering Genomes Produced by Recombinant Measles Viruses Are Recognized by RIG-I and LGP2 but Not MDA5. J. Virol..

[17] Baum A., Sachidanandam R., Garcia-Sastre A. (2010). Preference of RIG-I for short viral RNA molecules in infected cells revealed by next-generation sequencing. Proc. Natl. Acad. Sci. USA.

[18] Vasou A., Sultanoglu N., Goodbourn S., Randall R.E., Kostrikis L.G. (2017). Targeting Pattern Recognition Receptors (PRR) for Vaccine Adjuvantation: From Synthetic PRR Agonists to the Potential of Defective Interfering Particles of Viruses. Viruses.

[19] Gould P.S., Easton A.J., Dimmock N.J. (2017). Live Attenuated Influenza Vaccine contains Substantial and Unexpected Amounts of Defective Viral Genomic RNA. Viruses.

[20] Chaturvedi S., Vasen G., Pablo M., Chen X., Beutler N., Kumar A., Tanner E., Illouz S., Rahgoshay D., Burnett J. (2021). Identification of a therapeutic interfering particle-A single-dose SARS-CoV-2 antiviral intervention with a high barrier to resistance. Cell.

[21] Hein M.D., Arora P., Marichal-Gallardo P., Winkler M., Genzel Y., Pohlmann S., Schughart K., Kupke S.Y., Reichl U. (2021). Cell culture-based production and in vivo characterization of purely clonal defective interfering influenza virus particles. BMC Biol..

[22] Rand U., Kupke S.Y., Shkarlet H., Hein M.D., Hirsch T., Marichal-Gallardo P., Cicin-Sain L., Reichl U., Bruder D. (2021). Antiviral Activity of Influenza A Virus Defective Interfering Particles against SARS-CoV-2 Replication In Vitro through Stimulation of Innate Immunity. Cells.

[23] Xiao Y., Lidsky P.V., Shirogane Y., Aviner R., Wu C.T., Li W., Zheng W., Talbot D., Catching A., Doitsh G. (2021). A defective viral genome strategy elicits broad protective immunity against respiratory viruses. Cell.

[24] Sukhdeo S., Lee N. (2022). Influenza: Clinical aspects, diagnosis, and treatment. Curr. Opin. Pulm. Med..

[25] Paules C., Subbarao K. (2017). Influenza. Lancet.

[26] Labella A.M., Merel S.E. (2013). Influenza. Med. Clin. N. Am..

[27] Luo M. (2012). Influenza virus entry. Adv. Exp. Med. Biol..

[28] Arranz R., Coloma R., Chichon F.J., Conesa J.J., Carrascosa J.L., Valpuesta J.M., Ortin J., Martin-Benito J. (2012). The structure of native influenza virion ribonucleoproteins. Science.

[29] Jennings P.A., Finch J.T., Winter G., Robertson J.S. (1983). Does the higher order structure of the influenza virus ribonucleoprotein guide sequence rearrangements in influenza viral RNA?. Cell.

[30] Moeller A., Kirchdoerfer R.N., Potter C.S., Carragher B., Wilson I.A. (2012). Organization of the influenza virus replication machinery. Science.

[31] Te Velthuis A.J.W., Long J.C., Bauer D.L.V., Fan R.L.Y., Yen H.L., Sharps J., Siegers J.Y., Killip M.J., French H., Oliva-Martin M.J. (2018). Mini viral RNAs act as innate immune agonists during influenza virus infection. Nat. Microbiol..

[32] Finke S., Conzelmann K.K. (1999). Virus promoters determine interference by defective RNAs: Selective amplification of mini-RNA vectors and rescue from cDNA by a 3′ copy-back ambisense rabies virus. J. Virol..

[33] Rahim M.M.A., Parsons B.D., Price E.L., Slaine P.D., Chilvers B.L., Seaton G.S., Wight A., Medina-Luna D., Dey S., Grandy S.L. (2020). Defective Influenza A Virus RNA Products Mediate MAVS-Dependent Upregulation of Human Leukocyte Antigen Class I Proteins. J. Virol..

[34] Weis S., Te Velthuis A.J.W. (2021). Influenza Virus RNA Synthesis and the Innate Immune Response. Viruses.

[35] Turrell L., Lyall J.W., Tiley L.S., Fodor E., Vreede F.T. (2013). The role and assembly mechanism of nucleoprotein in influenza A virus ribonucleoprotein complexes. Nat. Commun..

[36] Perez J.T., Varble A., Sachidanandam R., Zlatev I., Manoharan M., Garcia-Sastre A., tenOever B.R. (2010). Influenza A virus-generated small RNAs regulate the switch from transcription to replication. Proc. Natl. Acad. Sci. USA.

[37] Vasilijevic J., Zamarreno N., Oliveros J.C., Rodriguez-Frandsen A., Gomez G., Rodriguez G., Perez-Ruiz M., Rey S., Barba I., Pozo F. (2017). Reduced accumulation of defective viral genomes contributes to severe outcome in influenza virus infected patients. PLoS Pathog..

[38] Saira K., Lin X., DePasse J.V., Halpin R., Twaddle A., Stockwell T., Angus B., Cozzi-Lepri A., Delfino M., Dugan V. (2013). Sequence analysis of in vivo defective interfering-like RNA of influenza A H1N1 pandemic virus. J. Virol..

[39] Lui W.Y., Yuen C.K., Li C., Wong W.M., Lui P.Y., Lin C.H., Chan K.H., Zhao H., Chen H., To K.K.W. (2019). SMRT sequencing revealed the diversity and characteristics of defective interfering RNAs in influenza A (H7N9) virus infection. Emerg. Microbes Infect..

[40] Frensing T., Pflugmacher A., Bachmann M., Peschel B., Reichl U. (2014). Impact of defective interfering particles on virus replication and antiviral host response in cell culture-based influenza vaccine production. Appl. Microbiol. Biotechnol..

[41] Duhaut S.D., Dimmock N.J. (1998). Heterologous protection of mice from a lethal human H1N1 influenza A virus infection by H3N8 equine defective interfering virus: Comparison of defective RNA sequences isolated from the DI inoculum and mouse lung. Virology.

[42] Hutchinson E.C., von Kirchbach J.C., Gog J.R., Digard P. (2010). Genome packaging in influenza A virus. J. Gen. Virol..

[43] Pelz L., Rudiger D., Dogra T., Alnaji F.G., Genzel Y., Brooke C.B., Kupke S.Y., Reichl U. (2021). Semi-continuous Propagation of Influenza A Virus and Its Defective Interfering Particles: Analyzing the Dynamic Competition to Select Candidates for Antiviral Therapy. J. Virol..

[44] Rodriguez A., Falcon A., Cuevas M.T., Pozo F., Guerra S., Garcia-Barreno B., Martinez-Orellana P., Perez-Brena P., Montoya M., Melero J.A. (2013). Characterization in vitro and in vivo of a pandemic H1N1 influenza virus from a fatal case. PLoS ONE.

[45] Noble S., Dimmock N.J. (1995). Characterization of putative defective interfering (DI) A/WSN RNAs isolated from the lungs of mice protected from an otherwise lethal respiratory infection with influenza virus A/WSN (H1N1): A subset of the inoculum DI RNAs. Virology.

[46] Laske T., Heldt F.S., Hoffmann H., Frensing T., Reichl U. (2016). Reprint of “Modeling the intracellular replication of influenza A virus in the presence of defective interfering RNAs. Virus Res..

[47] Isken B., Genzel Y., Reichl U. (2012). Productivity, apoptosis, and infection dynamics of influenza A/PR/8 strains and A/PR/8-based reassortants. Vaccine.

[48] Rabinowitz S.G., Huprikar J. (1979). The influence of defective-interfering particles of the PR-8 strain of influenza A virus on the pathogenesis of pulmonary infection in mice. J. Infect. Dis..

[49] Scott P.D., Meng B., Marriott A.C., Easton A.J., Dimmock N.J. (2011). Defective interfering virus protects elderly mice from influenza. Virol. J..

[50] Chen C., Fan W., Li J., Zheng W., Zhang S., Yang L., Liu D., Liu W., Sun L. (2018). A Promising IFN-Deficient System to Manufacture IFN-Sensitive Influenza Vaccine Virus. Front. Cell Infect. Microbiol..

[51] Garcia-Sastre A., Egorov A., Matassov D., Brandt S., Levy D.E., Durbin J.E., Palese P., Muster T. (1998). Influenza A virus lacking the NS1 gene replicates in interferon-deficient systems. Virology.

[52] Bdeir N., Arora P., Gartner S., Pohlmann S., Winkler M. (2021). Evidence that two instead of one defective interfering RNA in influenza A virus-derived defective interfering particles (DIPs) does not enhance antiviral activity. Sci. Rep..

[53] Ayaz S., Dibben O., Chapman D. (2021). Presence of defective viral genes in H1N1 live attenuated influenza vaccine strains is not associated with reduced human cell fitness or vaccine effectiveness. Vaccine.

[54] Tapia K., Kim W.K., Sun Y., Mercado-Lopez X., Dunay E., Wise M., Adu M., Lopez C.B. (2013). Defective viral genomes arising in vivo provide critical danger signals for the triggering of lung antiviral immunity. PLoS Pathog..

[55] Genoyer E., Lopez C.B. (2019). Defective Viral Genomes Alter How Sendai Virus Interacts with Cellular Trafficking Machinery, Leading to Heterogeneity in the Production of Viral Particles among Infected Cells. J. Virol..

[56] Kupke S.Y., Ly L.H., Borno S.T., Ruff A., Timmermann B., Vingron M., Haas S., Reichl U. (2020). Single-Cell Analysis Uncovers a Vast Diversity in Intracellular Viral Defective Interfering RNA Content Affecting the Large Cell-to-Cell Heterogeneity in Influenza A Virus Replication. Viruses.

[57] Martinez-Gil L., Goff P.H., Hai R., Garcia-Sastre A., Shaw M.L., Palese P. (2013). A Sendai virus-derived RNA agonist of RIG-I as a virus vaccine adjuvant. J. Virol..

[58] Baum A., Garcia-Sastre A. (2011). Differential recognition of viral RNA by RIG-I. Virulence.

[59] Hornung V., Ellegast J., Kim S., Brzozka K., Jung A., Kato H., Poeck H., Akira S., Conzelmann K.K., Schlee M. (2006). 5′-Triphosphate RNA is the ligand for RIG-I. Science.

[60] Pichlmair A., Schulz O., Tan C.P., Naslund T.I., Liljestrom P., Weber F., Reis e Sousa C. (2006). RIG-I-mediated antiviral responses to single-stranded RNA bearing 5′-phosphates. Science.

[61] Cui S., Eisenacher K., Kirchhofer A., Brzozka K., Lammens A., Lammens K., Fujita T., Conzelmann K.K., Krug A., Hopfner K.P. (2008). The C-terminal regulatory domain is the RNA 5′-triphosphate sensor of RIG-I. Mol. Cell.

[62] Schlee M., Roth A., Hornung V., Hagmann C.A., Wimmenauer V., Barchet W., Coch C., Janke M., Mihailovic A., Wardle G. (2009). Recognition of 5′ triphosphate by RIG-I helicase requires short blunt double-stranded RNA as contained in panhandle of negative-strand virus. Immunity.

[63] Kato H., Takeuchi O., Sato S., Yoneyama M., Yamamoto M., Matsui K., Uematsu S., Jung A., Kawai T., Ishii K.J. (2006). Differential roles of MDA5 and RIG-I helicases in the recognition of RNA viruses. Nature.

[64] Boergeling Y., Rozhdestvensky T.S., Schmolke M., Resa-Infante P., Robeck T., Randau G., Wolff T., Gabriel G., Brosius J., Ludwig S. (2015). Evidence for a Novel Mechanism of Influenza Virus-Induced Type I Interferon Expression by a Defective RNA-Encoded Protein. PLoS Pathog..

[65] Ayllon J., Garcia-Sastre A. (2015). The NS1 protein: A multitasking virulence factor. Curr. Top. Microbiol. Immunol..

[66] Killip M.J., Jackson D., Perez-Cidoncha M., Fodor E., Randall R.E. (2017). Single-cell studies of IFN-beta promoter activation by wild-type and NS1-defective influenza A viruses. J. Gen. Virol..

[67] Russell A.B., Elshina E., Kowalsky J.R., Te Velthuis A.J.W., Bloom J.D. (2019). Single-Cell Virus Sequencing of Influenza Infections That Trigger Innate Immunity. J. Virol..

[68] Wressnigg N., Shurygina A.P., Wolff T., Redlberger-Fritz M., Popow-Kraupp T., Muster T., Egorov A., Kittel C. (2009). Influenza B mutant viruses with truncated NS1 proteins grow efficiently in Vero cells and are immunogenic in mice. J. Gen. Virol..

[69] Ferko B., Stasakova J., Romanova J., Kittel C., Sereinig S., Katinger H., Egorov A. (2004). Immunogenicity and protection efficacy of replication-deficient influenza A viruses with altered NS1 genes. J. Virol..

[70] Moscona A. (1991). Defective interfering particles of human parainfluenza virus type 3 are associated with persistent infection in cell culture. Virology.

[71] Sarmiento R.E., Tirado R., Gomez B. (2002). Characteristics of a respiratory syncytial virus persistently infected macrophage-like culture. Virus Res..

[72] Baczko K., Liebert U.G., Billeter M., Cattaneo R., Budka H., ter Meulen V. (1986). Expression of defective measles virus genes in brain tissues of patients with subacute sclerosing panencephalitis. J. Virol..

[73] Calain P., Monroe M.C., Nichol S.T. (1999). Ebola virus defective interfering particles and persistent infection. Virology.

[74] Sidhu M.S., Crowley J., Lowenthal A., Karcher D., Menonna J., Cook S., Udem S., Dowling P. (1994). Defective measles virus in human subacute sclerosing panencephalitis brain. Virology.

[75] Calain P., Roux L., Kolakofsky D. (2016). Defective interfering genomes and Ebola virus persistence. Lancet.

[76] Rudiger D., Pelz L., Hein M.D., Kupke S.Y., Reichl U. (2021). Multiscale model of defective interfering particle replication for influenza A virus infection in animal cell culture. PLoS Comput. Biol..

[77] Zimmerman R.K., Nowalk M.P., Chung J., Jackson M.L., Jackson L.A., Petrie J.G., Monto A.S., McLean H.Q., Belongia E.A., Gaglani M. (2016). 2014–2015 Influenza Vaccine Effectiveness in the United States by Vaccine Type. Clin. Infect. Dis..

[78] Marcus P.I., Ngunjiri J.M., Sekellick M.J. (2009). Dynamics of biologically active subpopulations of influenza virus: Plaque-forming, noninfectious cell-killing, and defective interfering particles. J. Virol..

[79] Wang P., Zheng M., Lau S.Y., Chen P., Mok B.W., Liu S., Liu H., Huang X., Cremin C.J., Song W. (2019). Generation of DelNS1 Influenza Viruses: A Strategy for Optimizing Live Attenuated Influenza Vaccines. mBio.

[80] Lee A.C., Zhang A.J., Li C., Chen Y., Liu F., Zhao Y., Chu H., Fong C.H., Wang P., Lau S.Y. (2021). Intradermal vaccination of live attenuated influenza vaccine protects mice against homologous and heterologous influenza challenges. NPJ Vaccines.

[81] Ngunjiri J.M., Buchek G.M., Mohni K.N., Sekellick M.J., Marcus P.I. (2013). Influenza virus subpopulations: Exchange of lethal H5N1 virus NS for H1N1 virus NS triggers de novo generation of defective-interfering particles and enhances interferon-inducing particle efficiency. J. Interferon Cytokine Res..

[82] Mossler C., Groiss F., Wolzt M., Wolschek M., Seipelt J., Muster T. (2013). Phase I/II trial of a replication-deficient trivalent influenza virus vaccine lacking NS1. Vaccine.

[83] Talon J., Salvatore M., O’Neill R.E., Nakaya Y., Zheng H., Muster T., Garcia-Sastre A., Palese P. (2000). Influenza A and B viruses expressing altered NS1 proteins: A vaccine approach. Proc. Natl. Acad. Sci. USA.

[84] Hale B.G., Randall R.E., Ortin J., Jackson D. (2008). The multifunctional NS1 protein of influenza A viruses. J. Gen. Virol..

[85] Ngunjiri J.M., Ali A., Boyaka P., Marcus P.I., Lee C.W. (2015). In vivo assessment of NS1-truncated influenza virus with a novel SLSYSINWRH motif as a self-adjuvanting live attenuated vaccine. PLoS ONE.

[86] Ghorbani A., Ngunjiri J.M., Xia M., Elaish M., Jang H., Mahesh K.C., Abundo M.C., Jiang X., Lee C.W. (2019). Heterosubtypic protection against avian influenza virus by live attenuated and chimeric norovirus P-particle-M2e vaccines in chickens. Vaccine.

[87] Ghorbani A., Abundo M.C., Ji H., Taylor K.J.M., Ngunjiri J.M., Lee C.W. (2020). Viral Subpopulation Screening Guides in Designing a High Interferon-Inducing Live Attenuated Influenza Vaccine by Targeting Rare Mutations in NS1 and PB2 Proteins. J. Virol..

[88] Rathnasinghe R., Salvatore M., Zheng H., Jangra S., Kehrer T., Mena I., Schotsaert M., Muster T., Palese P., Garcia-Sastre A. (2021). Interferon mediated prophylactic protection against respiratory viruses conferred by a prototype live attenuated influenza virus vaccine lacking non-structural protein 1. Sci. Rep..

[89] Wacheck V., Egorov A., Groiss F., Pfeiffer A., Fuereder T., Hoeflmayer D., Kundi M., Popow-Kraupp T., Redlberger-Fritz M., Mueller C.A. (2010). A novel type of influenza vaccine: Safety and immunogenicity of replication-deficient influenza virus created by deletion of the interferon antagonist NS1. J. Infect. Dis..

[90] Nicolodi C., Groiss F., Kiselev O., Wolschek M., Seipelt J., Muster T. (2019). Safety and immunogenicity of a replication-deficient H5N1 influenza virus vaccine lacking NS1. Vaccine.

[91] Dimmock N.J., Easton A.J. (2014). Defective interfering influenza virus RNAs: Time to reevaluate their clinical potential as broad-spectrum antivirals?. J. Virol..

[92] Meng B., Bentley K., Marriott A.C., Scott P.D., Dimmock N.J., Easton A.J. (2017). Unexpected complexity in the interference activity of a cloned influenza defective interfering RNA. Virol. J..

[93] Bdeir N., Arora P., Gartner S., Hoffmann M., Reichl U., Pohlmann S., Winkler M. (2019). A system for production of defective interfering particles in the absence of infectious influenza A virus. PLoS ONE.

[94] Dimmock N.J., Rainsford E.W., Scott P.D., Marriott A.C. (2008). Influenza virus protecting RNA: An effective prophylactic and therapeutic antiviral. J. Virol..

[95] Dimmock N.J., Dove B.K., Scott P.D., Meng B., Taylor I., Cheung L., Hallis B., Marriott A.C., Carroll M.W., Easton A.J. (2012). Cloned defective interfering influenza virus protects ferrets from pandemic 2009 influenza A virus and allows protective immunity to be established. PLoS ONE.

[96] Dimmock N.J., Dove B.K., Meng B., Scott P.D., Taylor I., Cheung L., Hallis B., Marriott A.C., Carroll M.W., Easton A.J. (2012). Comparison of the protection of ferrets against pandemic 2009 influenza A virus (H1N1) by 244 DI influenza virus and oseltamivir. Antivir. Res..

[97] Kupke S.Y., Riedel D., Frensing T., Zmora P., Reichl U. (2019). A Novel Type of Influenza A Virus-Derived Defective Interfering Particle with Nucleotide Substitutions in Its Genome. J. Virol..

[98] Hein M.D., Kollmus H., Marichal-Gallardo P., Puttker S., Benndorf D., Genzel Y., Schughart K., Kupke S.Y., Reichl U. (2021). OP7, a novel influenza A virus defective interfering particle: Production, purification, and animal experiments demonstrating antiviral potential. Appl. Microbiol. Biotechnol..

[99] Zhao H., To K.K.W., Chu H., Ding Q., Zhao X., Li C., Shuai H., Yuan S., Zhou J., Kok K.H. (2018). Dual-functional peptide with defective interfering genes effectively protects mice against avian and seasonal influenza. Nat. Commun..

[100] Zhao H., Zhang C., Lam H., Meng X., Peng Z., Yeung M.L., Chan J.F., Kai-Wang To K., Yuen K.Y. (2022). Peptidic defective interfering gene nanoparticles against Omicron, Delta SARS-CoV-2 variants and influenza A virus in vivo. Signal Transduct. Target Ther..

[101] Easton A.J., Scott P.D., Edworthy N.L., Meng B., Marriott A.C., Dimmock N.J. (2011). A novel broad-spectrum treatment for respiratory virus infections: Influenza-based defective interfering virus provides protection against pneumovirus infection in vivo. Vaccine.

